# Effect of C/N Ratio on the Removal of Nitrogen and Microbial Characteristics in the Water Saturated Denitrifying Section of a Two-Stage Constructed Rapid Infiltration System

**DOI:** 10.3390/ijerph15071469

**Published:** 2018-07-12

**Authors:** Qinglin Fang, Wenlai Xu, Gonghan Xia, Zhicheng Pan

**Affiliations:** 1 State Key Laboratory of Geohazard Prevention and Geoenvironment Protection, Chengdu University of Technology, Chengdu 610059, China; xiaofang20111009@126.com; 2 State Environmental Protection Key Laboratory of Synergetic Control and Joint Remediation for Soil & Water Pollution, Chengdu University of Technology, Chengdu 610059, China; txgsfy@163.com; 3 Haitian Water Grp Co Ltd., Chengdu 610059, China; pan12487616@126.com

**Keywords:** two-stage constructed rapid infiltration system, water-saturated denitrifying section, C/N ratio, denitrifying bacteria, extracellular polymeric substances

## Abstract

The aim of this study was to improve the removal of nitrogen pollutants from artificial sewage by a modeled two-stage constructed rapid infiltration (CRI) system. The C/N ratio of the second stage influent was elevated by addition of glucose. When the C/N ratio was increased to 5, the mean removal efficiency of total nitrogen (TN) reached up to 75.4%. Under this condition, the number of denitrifying bacteria in the permanently submerged denitrifying section (the second stage) was 22 times higher than that in the control experiment without added glucose. Elevation of the C/N ratio resulted in lower concentrations of nitrate and TN in the second stage effluent, without impairment of chemical oxygen demand removal. The concentration of nitrate and TN in effluent decreased as the abundance of denitrifying bacteria increased. Moreover, the bacterial biofilms that had formed in the sand of the second stage container were analyzed. The secretion of extracellular polymeric substances, a major constituent of biofilms, was enhanced as a result of the elevated C/N ratio, which lead to the improved protection of the bacteria and enhanced the removal of pollutants.

## 1. Introduction

The constructed rapid infiltration system (CRI) is a novel ecologically friendly technology for sewage treatment based on a modification of conventional sewage treatment [1]. The core of a CRI is a mixture of river sand and gravel that replaces conventional soil layers as the main infiltration media, which improves hydraulic load [2]. In addition, a dry-wet cycling of water feeding and draining is applied, which alternates the operation mode between an aerobic and (facultative) anaerobic environment, thus employing a more diverse mix of microorganisms. The unique operation mode and the rich diversity of microorganisms colonizing the filling medium of a CRI are favorable for efficient removal of pollutants from sewage [3].

**Figure 1:**
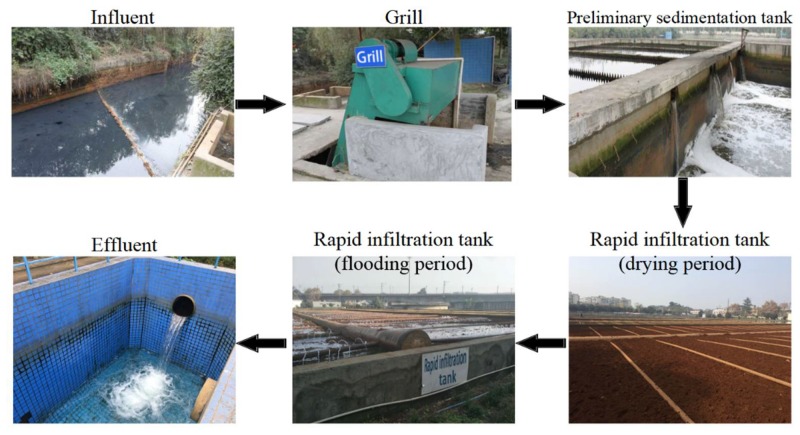
Practical engineering of the Phoniex River CRI system operated successfully for 12 years in Chengdu, China.

The common practice, with photographs shown in Figure 1, results in satisfying removal performances of organic pollutants and suspended solids (SS); in particular removal rates of chemical oxygen demand (COD) of 85% can be established, while ammonia nitrogen (NH_4_^+^-N) removal rates can be as high as 90%. In contrast, removal of total nitrogen (TN) is relatively poor, only reaching 10–30% [4]. In a simulation experiment, Wang et al. [5] found that the upper part of the CRI simulation column contained higher amounts of denitrifying bacteria, related to presence of organic carbon sources, better oxygen transmission and aeration, while these were lacking in the anaerobic environment of the lower part of the CRI simulation column. Due to the limitation of denitrifying bacteria and organic carbon sources in that lower part, the overall removal of TN from the effluent was poor [5]. Removal of TN, the sum of NH_4_^+^-N, NO_3_^−^-N, NO_2_^−^-N and organic nitrogen, is difficult due to fluctuations between these various nitrogen forms. Insufficient removal and excessive discharge of TN into the environment can lead to eutrophication of rivers and lakes, which poses a world-wide environmental problem [6]. Thus, various methods have been employed to improve the nitrogen removal efficiency of a CRI. It was shown that the main factors determining nitrogen removal in a CRI are the concentration of dissolved oxygen, the ratio of C/N in the water, and the residence time of NO_3_^−^-N in the anaerobic section [7]. To improve nitrogen removal performance, a sub-section of the effluent can be fed back into the CRI, which in simulated columns resulted in a 64.8% increase of TN removal efficiency [8]. Matsumoto [9] observed that denitrification by CRI could be improved when the C/N ratio in the influent was increased to 2. Moreover, Chen et al. [10] found that the TN removal rate could reach over 60% by adding 7 mg/L Fe^3+^ to the water-saturated section of CRI. Lastly, Song et al. [11] added corncob waste to the water-saturated section of CRI, which increased the nitrogen removal rate to above 79%. Thus, various methods exist to improve the nitrogen removal efficiency of CRI systems, but few studies determined the optimal C/N ratio in influent of the water-saturated section, or the variation in microbial biomass related to changes in C/N ratio in that section.

**Figure 2:**
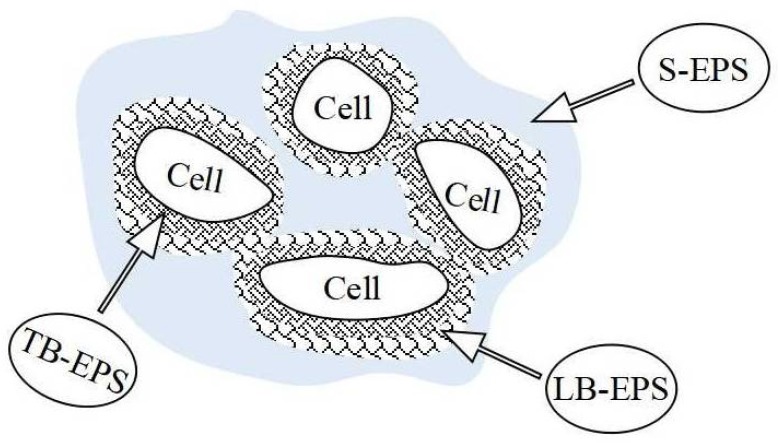
Schematic structure of EPS composition.

The microorganisms active in CRI system mostly form biofilms, and the necessary aerobic, anaerobic and facultative anaerobic processes for efficient water purification depend on large numbers of microbes attached to the surface of the filling medium [12]. As an important component of biofilms, extracellular polymeric substances (EPS) constitute over 80% of biofilm organic matter [13]. These substances form a macromolecular gelatinous matrix with a three-dimensional network structure mainly made up of proteins and carbohydrates [14]. The overall biofilm community has been described as a “microbial city” [15], whereby the EPS forms the “accommodation” for the microbial cells [16]. EPS that can easily be released into solution is described as the soluble EPS (S-EPS) fraction; EPS that remains bound to the bacteria (bound EPS or B-EPS) can be further divided into loosely bound EPS (LB-EPS) and tightly bound EPS (TB-EPS, Figure 2). Due to its typical physico-chemical properties, EPS enables a close packing of microbial cells, which protects them from external adverse effects. Moreover, EPS can serve as an energy and carbon source for the microbes after it is degraded into smaller molecules by extracellular enzymes. Lastly, EPS plays an important role in promoting microbial cell aggregation, as it accelerates biofilm formation and maintains collective structures. Due to these effects, EPS improves CRI performance, and has become the focus of research towards biological sewage treatment [17]. For instance, a positive correlation was found between the EPS content in sludge and its sludge volume index in a sequential batch reactor (SBR) [18]. The concentration of proteins and carbohydrates in LB-EPS of activated sludge correlated with the C/N ratio in influent [19], although in those studies the EPS concentration of the biofilms in the water-saturated section of CRI had not been reported.

The goal of the current study was to use simulation columns of a two-stage CRI system to study the nitrifying simulation column (the first stage) and the water-saturated denitrifying simulation column (the second stage) separately, during treatment simulation of artificial sewage. The effect of variable C/N ratios in the second-stage influent on pollutant removal was determined, and the microbial community and biofilm EPS concentrations were quantified in the water-saturated denitrifying section. This addressed the influence of different C/N ratios in influent on the pollutant removal performance of CRI from a microcosmic perspective, which provides insights to improve nitrogen removal.

## 2. Materials and Methods

### 2.1. Experimental Design

**Figure 3:**
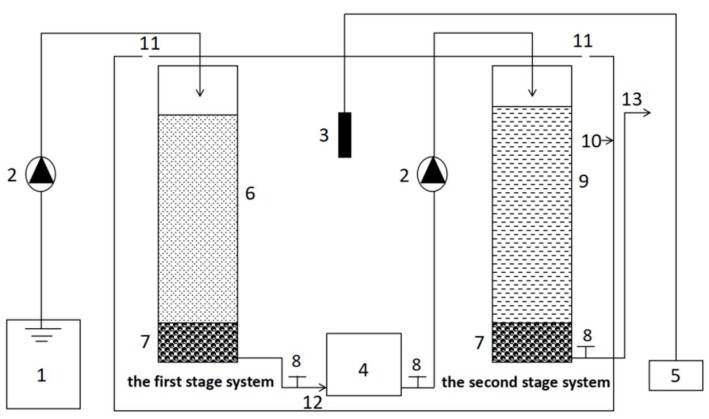
Schematic diagram of the simulated two-stage column CRI. 1: Feeding tank (V = 5 L); 2: Peristaltic pump; 3: Thermometer; 4: Tank to add a carbon source (V = 1 L); 5: Temperature controller; 6: Filling medium; 7: Support layer; 8: Valve; 9: Saturated section; 10: Insulation mantle; 11: Air hole; 12: Effluent outlet of the first stage; 13: Effluent outlet of the second stage.

A schematic diagram of the simulation columns representing the two stages of the CRI system used in this study is shown in Figure 3. The model is composed of two parts: a nitrifying simulation column (the first stage) and a water-saturated denitrifying simulation column that represents the second stage; these were connected in series. The temperature of the columns was kept constant at 30 ± 1.2 °C by means of a temperature-controlled insulation mantle. The columns were made of polyvinyl chloride (PVC) with a diameter of 8 cm and a height of 30 cm. The filling medium consisted of two layers: a 4 cm high supporting layer of pebbles (5.0–10.0 mm) mixed with gravel (3.0–4.0 mm) was covered by a 21 cm high treatment layer of 90% river sand (0.25–0.30 mm grain size), mixed with 5% marble sand (1.0–2.0 mm) and 5% zeolite sand (1.5–1.7 mm). The sampling outlet of the first stage column was positioned 1 cm above the bottom of the column, while the sampling outlet of the second-stage column was fitted at a height of 26 cm. In this way, the second stage remained continuously submerged. The influent sewage was pumped up so that it entered from the top of the column, moved through the packing medium vertically, and left by the outlet where the water quality was measured. In order to regulate the C/N ratio of the influent of the second stage, a tank with a volume of 1 L was installed between the columns, from which a carbon source could be added.

### 2.2. Sewage and Operational Conditions

The influent used in this study was synthetic sewage made up of glucose, sodium acetate, ammonium sulfate, ammonium chloride, potassium phosphate, sodium carbonate and peptone, which was refilled every three days. The water quality parameters are shown in Table 1. The sewage influent entered the first stage column via a dry-wet alternating operation mode, with water feeding with a hydraulic load of 0.6 m^3^·(m^−3^·d^−1^), each feeding time would last for 1.5 h and this was performed twice every 24 h, followed by a drying time lasting 10.5 h, and a water flow of 200 mL/h. The second-stage column received the effluent of the first stage column as influent. This was added by intermittent feeding using the same regime as for the first stage column.

### 2.3. Batch Experiments

To study the effect of different C/N ratios of the second stage influent, three experimental setups were constructed as described in Section 2.2 and these were started under the same conditions, to give Test 1, Test 2 and Test 3 (T1, T2, T3). After operation for 30 days, the removal percentages of COD and NH_4_^+^-N from effluent were all stabilized, reaching levels up to 90%, indicating that biofilms had formed successfully in the filling medium [8]. From this time point onwards, T1 served as control treatment not receiving additives; the C/N ratio in the second stage influent of T2 was increased by adding a carbon source at twice the amount of COD per nitrate-nitrogen (C/N 2:1) and that of T3 was increased to a C/N ratio of 5:1. Glucose was used for this, as it is cheap, easy to obtain, non-toxic, and easily biodegraded [20]. Influent and effluent of all three experiments was sampled every two days and used for analysis. At the end of the experiment, after the removal efficiency of TN in the second stage effluent of all experimental tests had stabilized, the filling medium (sand) was sampled from the second stage column for analysis.

### 2.4. Analytical Methods

#### 2.4.1. Water Quality Analytical Methods

The concentration of COD in the water was determined using the potassium dichromate method, the TN concentration was measured by UV spectrometry and the concentration of NH_4_^+^-N was determined by the Nessler’s reagent colorimetric method. Lastly, NO_3_^−^-N was measured by UV spectrometry, using standard procedures [21].

#### 2.4.2. Microbiological Analysis and EPS Quantization 

Bacterial counts of nitrifying and denitrifying bacteria were determined by the most probable number method (MPN) [22]. For this, 10 g of sand was collected from the position 10 cm above the supporting layer of the water-saturated denitrifying column and this was added to 100 mL sterile water in conical flasks that were shaken for 30 min. The suspension was then serially diluted by 10-fold steps and this dilution series was inoculated into culture medium and cultured for 14 days at 28 °C. Finally, the culture medium was titrated by a chromogenic agent, and the number of bacteria was obtained by comparing the number of tubes in the chromogenic medium with the MPN value table.

EPS in biofilms was extracted as previously described [14,23]. Sand samples (10 g) were mixed with 45 mL extraction buffer (2 mmol/L Na_3_PO_4_, 4 mmol/L NaH_2_PO_4_, 9 mmol/L NaCl, 1 mmol/L KCl, pH 7.0), treated by ultrasonication for 5 min and centrifuged (20 min 2000× *g*). The supernatant was filtered through a milipore filter of 0.45 µm to give the extracted S-EPS fraction. The pellet was resuspended into 45 mL extraction buffer and shaken for 1 h after which centrifugation was performed at 5000× *g* for 20 min. The filtered supernatant from this second centrifugation step resulted in LB-EPS, while the resuspended pellet was heated to 60 °C for 1 h and centrifuged at 10,000× g (20 min). The supernatant of this third centrifugation step was filtered as above to give TB-EPS.

The amount of biofilm in the sand samples was estimated as follows. The samples were briefly rinsed in distilled water and then mixed with 1 M NaOH and incubated at 70 °C for 30 min. Following ultrasonic oscillation for 15 min the biofilm containing extracts were filtered (0.45 µm) using pre-weighed membranes. After filtering, the membranes were dried in an oven at 103 °C for 1 h. The amount of biofilm was estimated by the difference in weight of the membrane filters before and after drying (SS).

The content of protein and carbohydrates was used to characterize EPS content (mg/g SS) as these are the main components of EPS [14]. The carbohydrate content was measured by means of the phenol-sulfuric method [24] with glucose as the standard. The protein content was measured by Coomassie brilliant blue [18] using bovine serum albumin as the standard.

## 3. Results and Discussion 

### 3.1. Effect of C/N Ratio on Removal Efficiency of Ammonium and Nitrate

In the first stage of the experimental CRI, ammonium is converted to nitrate according to Equation (1):

(1) $$NH_{4}{}^{+}+2O_{2}=NO_{3}{}^{-}+H_{2}O+2H^{+}$$

**Figure 4:**
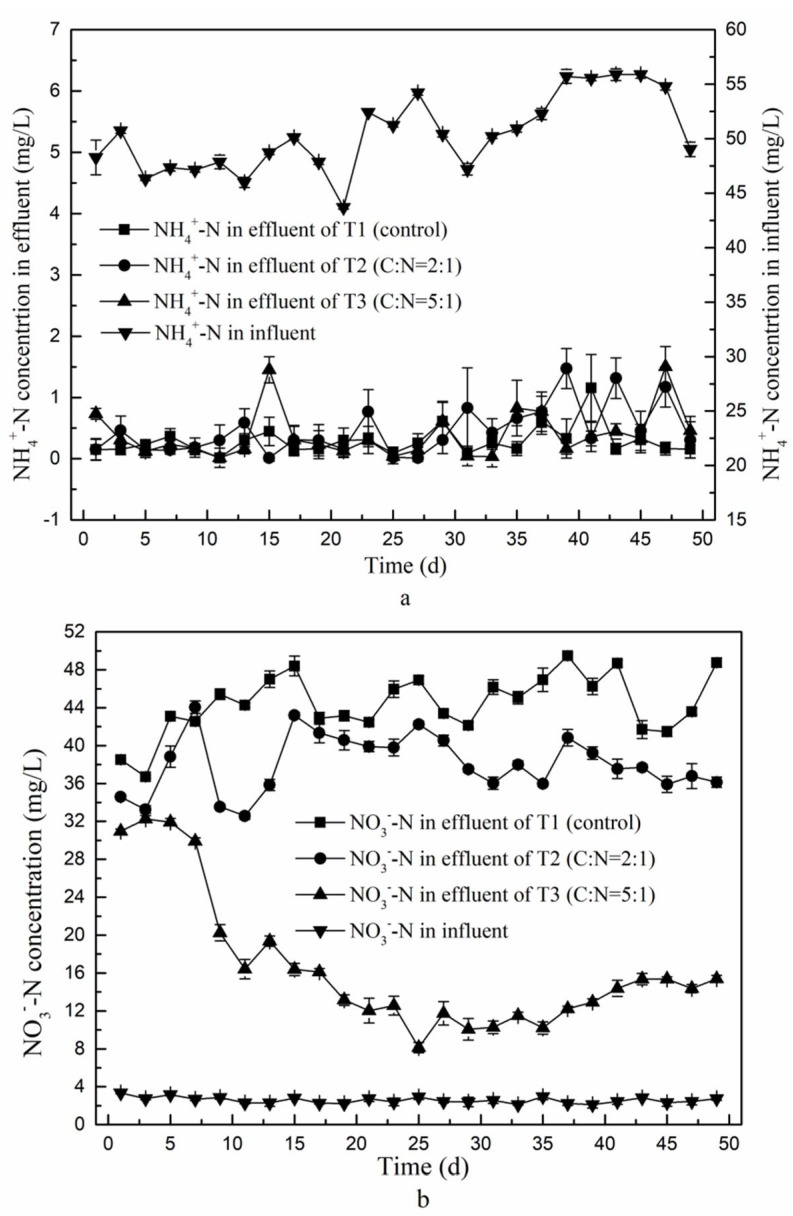
Overall ammonium and nitrate concentrations of a modeled two-stage CRI over time. (**a**) Absolute concentrations of NH_4_^+^-N in first-stage influent and in the second-stage effluent of the three experimental setups; (**b**) The nitrate concentration in influent of the first stage and in the effluent of the second stage in the three experimental setups.

The concentrations and removal efficiencies of ammonium in the effluent of the second stage over time was determined for T1 (control), T2 (glucose added at a ratio of 2:1, C/N), and T3 (C/N increased to 5:1). The results are shown in Figure 4a. The relative removal efficiency was calculated as the difference in concentration between the first-stage influent and the second-stage effluent divided by the concentration in the first stage influent.

The mean concentration of ammonium in the second stage effluent was lower in the control T1 (0.3 mg/L), than in T2 and T3 (0.4 mg/L and 0.5 mg/L, respectively). The removal percentages of NH_4_^+^-N reached up to 99.1% with only minor differences between the three experiments, indicating that the modeled CRI resulted in good overall ammonium removal and an increased C/N ratio in the second-stage influent contributed little to improve this. The removal of NH_4_^+^-N in this CRI mainly occurs through adsorption and nitrification, which take place in the first stage, so that alterations in conditions of the second stage have little effect. Moreover, as a result of the used setup, the denitrifying column provides a constant submerged environment, which restricts the nitrification reaction [5] that would otherwise convert ammonium to nitrate (Equation (1)). As a result, varying the carbon to nitrogen ratio in the influent of the second phase has no effect on the overall removal rate of ammonium.

The nitrate in the effluent is mainly generated by nitrifying bacteria that oxidize ammonium during the dry phase [25]. As shown in Figure 4b, the mean concentration of NO_3_^−^-N in the first-stage influent was only around 2.7 mg/L, but it was much higher in the effluent of the second stage. In the control the mean nitrate concentration reached 45 mg/L in second-stage effluent, accounting for 93% of the TN concentration. Thus, the nitrifying section of the CRI resulted in nearly complete nitrification of the available nitrogen, as a result of the employed dry-wet alternating operation mode. Since negatively charged nitrate is not adsorbed by dielectric particles that are mostly also negatively charged [26], it remained in solution and was efficiently flushed out with the water flow, resulting in high concentration of nitrate and TN in the effluent. Wang et al. [25] showed that an extension of the residence time of the denitrifying section could improve the denitrification capacity of a CRI, as it would allow bacteria more time for nitrogen conversion. We extended the retention time to 6.5 h in the second stage by constructing a permanent water-saturated environment. However, this did not significantly increase the removal efficiency of nitrate in second-stage effluent of T1. Hou et al. [27] reported that a C/N ratio less than 2 in influent provided insufficient amounts of carbon for denitrification, resulting in low TN removal in effluent. We found that an increase in C/N to 2:1 in T2 resulted in average nitrate concentrations in the second-stage effluent of 38.6 mg/L, which represents a decrease of 6.4 mg/L compared to the control T1. With the highest tested C/N ratio of 5:1 (in T3) the nitrate concentration decreased by 71.8% compared to T1, so that only 12.7 mg/L NO_3_^−^-N remained in the second stage effluent. Thus, we confirmed that the denitrifying capacity of a CRI can be improved by adding an external carbon source to the water-saturated denitrifying section, in order to meet the energy demand of denitrifying bacteria.

### 3.2. Effect of C/N Ratio on Removal Efficiency of Total Nitrogen

**Figure 5:**
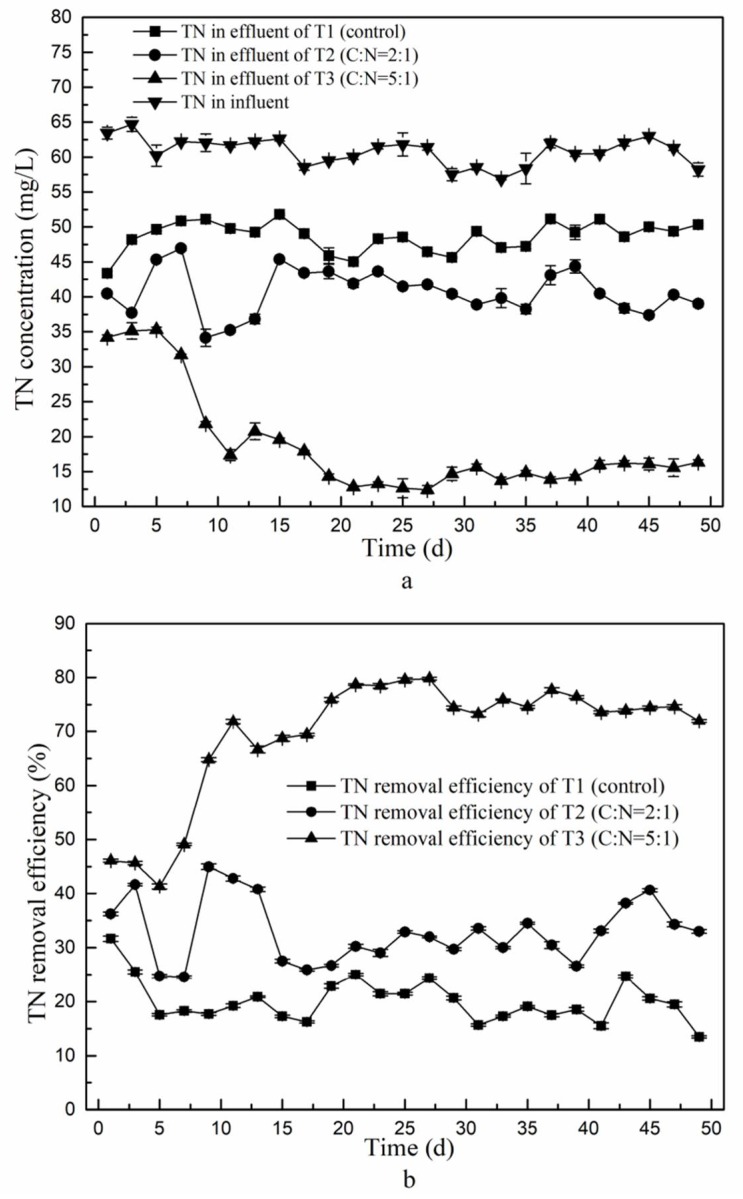
Total nitrogen removal of the second stage of the CRI of the three experimental tests with variable C/N ratios. (**a**) Total nitrogen concentration in influent of the first stage and in effluent of the second stage; (**b**) Total nitrogen removal efficiency.

CRI has been shown to perform well for removal of COD, NH_4_^+^-N and SS, but under standard conditions the removal of TN is typically only around 10–30% [4]. This can be improved by an increased C/N ratio in the second stage influent, as shown in Figure 5.

Initially, the TN concentrations in the second-stage effluent of all three experiments were above 35 mg/L during the first 5 days (Figure 5a), which indicates a relatively poor nitrogen removal performance at this early stage of the experiments. Possibly, the denitrifying bacteria in the second phase need time to adapt to the new conditions applying after addition of an external carbon source. From day 9 onwards, the TN concentration in the second stage effluent of T2 and T3 decreased, to 34.2 mg/L and 21.9 mg/L respectively. As the experiments continued, this TN concentration in effluent of T3 kept declining, but in T2 it increased again. This indicates that the C/N ratio of 2:1 enhanced the denitrifying capacity of the second stage section to a certain extent by promoting growth of denitrifying bacteria, but it was insufficient to meet the increasing demand of carbon as the denitrifying bacteria proliferated. This is in line with findings reported by Fan et al. [28], who tested a C/N ratio of 2.5 in influent of a constructed wetland which resulted in a TN removal efficiency of only around 25%.

On day 17 of the experiments, the TN concentration in the second stage effluent of was more or less stable with mean TN concentrations in the second stage effluent of 48.4 mg/L (giving a 19.6% removal rate) in T1, 41.0 mg/L (31.8% removal rate) in T2 and 14.7 mg/L (75.4% removal rate) in T3. The TN removal rate of the second stage effluent had thus nearly doubled in T2 compared to T1, but the resulting concentration was still too high to meet the national sewage discharge B standard (GB18918-2002) (TN ≤ 20 mg/L). The second-stage effluent of T3 passed this standard, and even passed the sewage discharge a standard (TN ≤ 15 mg/L). The TN removal of T3 had improved by 69.5% compared to T1. This clearly shows that addition of a carbon source in the second stage influent enhanced the nitrogen removal efficiency of the system, whereby a C/N ratio of 5 performed better than a ratio of 2. The best TN performance obtained resulted in 79.8% removal of TN.

### 3.3. Effect of C/N Ratio on Removal Efficiency of Chemical Oxygen Demand

**Figure 6:**
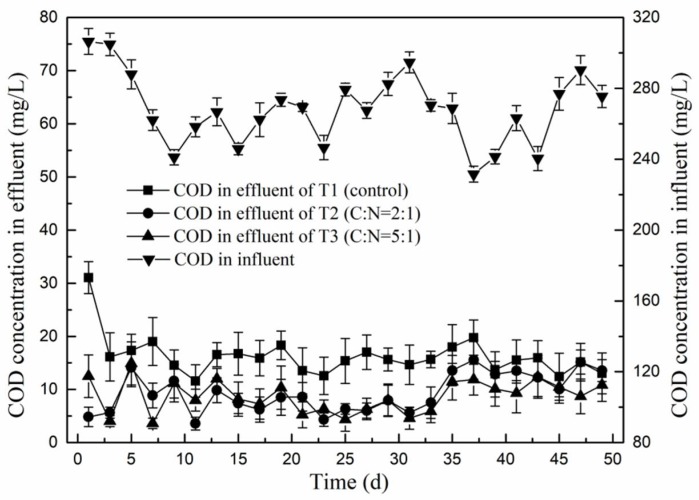
The chemical oxygen demand (COD) concentration in influent of the first stage and in effluent of the second stage of the three experimental setups.

The COD concentration of the first stage influent was 268.3 ± 20.0 mg/L. This reduced during the first stage to 22.8 mg/L, 24.5 mg/L and 20.5 mg/L for effluent of T1, T2 and T3 respectively, so the mean fraction of removed COD reached up to 90% in all three experiments. However, the mean concentration of NO_3_^−^-N in the first stage effluent remained above 48.3 mg/L, resulting in a C/N ratio in the first stage effluent below 1. The effect of increasing this ratio to 2 and 5 in T2 and T3, respectively, on COD removal is shown in Figure 6. Whereas the mean concentration of COD in the second stage effluent of T1 decreased to only 16.2 mg/L, for T2 and T3 it went down to 9.3 mg/L and 8.7 mg/L respectively. Compared with T1, the mean COD concentration in the second stage effluent of T2 and T3 were much lower, indicating that the addition of glucose to the second stage influent had not resulted in an increase of COD in the effluent, but rather promoted the removal of COD during the second stage. Clearly, this external carbon source enhanced the activity and growth of denitrifying bacteria. The amount of glucose required for this was not fully met under experimental condition T2, as the best COD removal was obtained with T3, which received the highest amount of glucose. In another study, Chen et al. [7] found that the removal efficiency of COD could reach up to 85% in the nitrifying section of CRI, while Wang et al. [20] determined an optimal C/N ratio for denitrification of 6–7 when they used glucose as external carbon source. Zhang [29] noticed that nitrogen could be completely removed in SBR by adjusting the C/N ratio to 7.1 with added glucose. Yan et al. [30] reported that if the main purpose of the sewage treatment was to remove nitrogen, the C/N ratio in influent should be slightly lower than the optimum C/N ratio, which could not only guarantee high COD removal rates (resulting in low residual organic matter), but also produce a better nitrogen removal performance.

### 3.4. Analysis of the Microbial Communities and EPS

**Figure 7:**
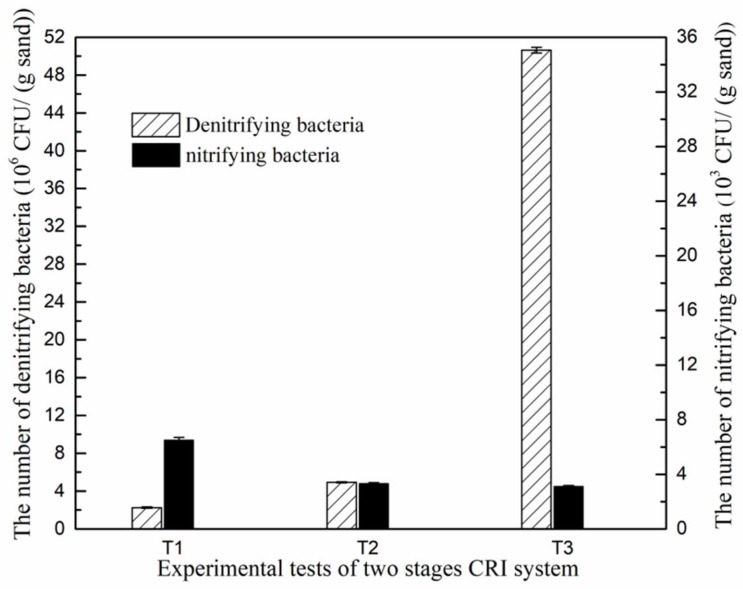
The number of denitrifying bacteria (10^6^ CFU·(g sand)^−1^) and nitrifying bacteria (10^3^ CFU·(g sand)^−1^) in the sand of the water-saturated denitrifying section.

The bacteria in the water-saturated denitrifying section were quantitatively analyzed by the MPN method (Figure 7). The sand from the second stage of T1 contained 6.5 × 10^3^ CFU (g sand)^−1^ nitrifying bacteria, and approximately half were present in the sand of T2 and T3 (3.3 × 10^3^ and 3.1 × 10^3^ CFU·(g sand)^−1^, respectively). The low amount of dissolved oxygen and NH_4_^+^-N in the fully submerged second stage most probably limited the growth of these aerobic bacteria [31]. These relatively low counts of nitrifying bacteria support the conclusion that the nitrification reaction was restricted in the fully submerged phase of the CRI. A previous study [32] described that when organic matter was abundant in sewage, heterotrophic bacteria could grow rapidly and these would outcompete nitrifying bacteria for dissolved oxygen and nutrients.

The numbers of denitrifying bacteria were a factor of 1000 higher, in the order of 10^6^–10^7^ CFU·(g sand)^−1^, In particular in T3 their numbers peaked to 5.1 × 10^7^ CFU·(g sand)^−1^, which was over 20 times higher than in T1, and 10 times higher than in T2. Therefore, under the water-saturated conditions applied, an increase of the C/N ratio in the influent promoted the proliferation of denitrifying bacteria as long as the nitrogen supply was sufficient. When the C/N ratio increased from 2 to 5, the number of denitrifying bacteria increased by more than one order of magnitude. Combining all results, it was found that the concentrations of TN and NO_3_^−^-N in the second stage effluent decreased with an increase in numbers of denitrifying bacteria in the water-saturated denitrifying section. This suggests that the number of denitrifying bacteria presenting this section of the CRI is animportant factor determining the nitrogen removal performance, and this can be influenced by changing the C/N ratio. However, the specific relationship between the bacterial abundance and the nitrogen removal performance needs to be further explored.

**Figure 8:**
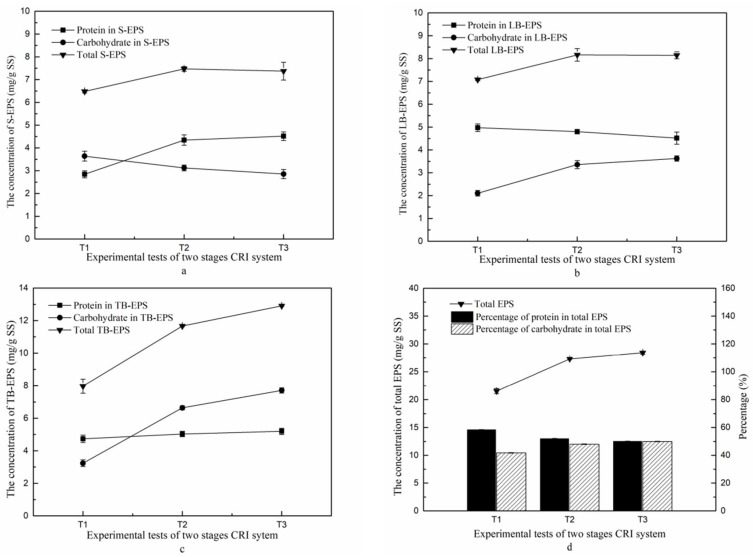
The concentrations of EPS (triangles), and its protein (squares) and carbohydrate (circles) fractions (all expressed as mg/g SS) in the sand of the second stage system. (**a**) S-EPS; (**b**) LB-EPS; (**c**) TB-EPS; (**d**) Total EPS. The percentages of protein and carbohydrate fractions in total EPS are also shown as bars in (**d**).

The amount of S-EPS, LB-EPS and TB-EPS in the formed biofilms was determined for the denitrifying section and the total EPS concentration was calculated as the sum of these fractions. The concentrations of proteins and carbohydrates were also determined, with results shown in Figure 8. Panels 8a and 8b show that the amount of biofilm S-EPS and LB-EPS was higher in T2 than in T1, probably as a result of the increased C/N ratio, though a further increase of this ratio in T3 did not elevate S-EPS and LB-EPS further. Their concentrations all remained below 10 mg/g SS. In contrast, the fraction of TB-EPS (panel 8c) was higher in T2 than in T1, and higher still in T3, with concentrations exceeding 10 mg/g SS. The total EPS reached nearly 30 mg/g SS in T3 (panel 8d). This increase in biofilm EPS as a result of the increase by C/N ratio was mostly attributed to TB-EPS, confirming that TB-EPS is the key factor affecting the concentration of total EPS [19].

The comparisons of protein and carbohydrate fractions of EPS between T1, T2 and T3 produced a less clear picture, with an increase for proteins as a result of increased C/N ratio in S-EPS and TB-EPS but not in LB-EPS. For the carbohydrate fraction, the trends were in the opposite direction (Figure 8). The reason for these observations may be that as long as the nitrogen source was sufficient in the influent, addition of extra glucose could promote the formation of protein in S-EPS, but this limited the production of carbohydrates due to competition. The fractions of both protein and carbohydrate were increased in TB-EPS when the C/N ratio was increased (Figure 8c), which indicated that the increase of carbon source could simultaneously promote the increase of protein and carbohydrate concentrations in biofilm TB-EPS. Moreover, the contents of protein and carbohydrate in total EPS (Figure 8d) showed the same changing trends as those of TB-EPS, which indicates that increasing the C/N ratio in the second stage influent was favorable to enhance the secretion of protein and carbohydrates in total EPS. This would lead to an improved ability of biofilm formation, which would protect the microbial cells and allow more efficient pollutants removal.

It has been reported that the protein fraction of EPS could promote biofilm and granular sludge formation, and maintain the stability of microbial aggregates [33]. The carbohydrate fraction serves as the skeleton of these microbial aggregates, and when a mutation was introduced in a gene that abolishes carbohydrate biosynthesis, the bacteria could no longer form mature biofilms [34]. However, the contents of protein and carbohydrate in biofilm LB-EPS both showed a opposite trend to that of biofilm S-EPS, which may be because the carbon source was primarily used to generate carbohydrate and protein, which was then gradually degraded and utilized by the microorganisms when the carbon source became restricted over time. 

## 4. Conclusions

This work shows that the removal efficiency of TN by a two-stage CRI could reach 75.4 ± 2.8%, provided the C/N ratio in the influent of the second stage was elevated to 5 by the addition of glucose. We have shown that elevating the C/N ratio can promote the removal of nitrate and TN in the second-stage effluent, without impairing the COD removal. The quantity of denitrifying bacteria in the sand of the permanently submerged denitrifying section increased as a result of the higher C/N ratio, and the concentrations of NO_3_^−^-N and TN in the subsequent effluent both decreased as the quantity of denitrifying bacteria increased. Moreover, analysis of the bacterial biofilms that had formed in the sand of the denitrifying section showed that the elevated C/N ratio had enhanced secretion of the total biofilm EPS, which leads to improved biofilm formation and enhances the pollutant’s removal in the two-stage CRI.

## Figures and Tables

**Table 1:** Water quality parameters of influent of the first stage.

Water Quality Parameters	Mean Concentration (mg/L)
Chemical Oxygen Demand (COD)	268.3 ± 20.0
NH_4_^+^-N	50.4 ± 3.5
NO_3_^−^-N	2.6 ± 0.3
NO_2_^−^-N	0.039 ± 0.06
Total nitrogen (TN)	60.8 ± 2.0
pH	8.2
